# The road towards protection of all against tetanus

**DOI:** 10.1371/journal.pntd.0011611

**Published:** 2023-09-21

**Authors:** Md. Abdullah Saeed Khan, Mohammad Jahid Hasan, Mohammad Delwer Hossain Hawlader

**Affiliations:** 1 Tropical Disease and Health Research Centre, Dhaka, Bangladesh; 2 National Institute of Preventive and Social Medicine, Dhaka, Bangladesh; 3 North South University, Dhaka, Bangladesh; Yale University School of Medicine, UNITED STATES

## Abstract

In low- and middle-income countries (LMICs), tetanus continues to be a major public health concern. Although vaccination campaigns have been effective in lowering the incidence of tetanus worldwide, some areas continue to experience a considerable number of cases and fatalities. Adult tetanus is frequently underreported because there is insufficient systematic surveillance and reporting. A high proportion of tetanus patients die because of a lack of adequate critical care services, particularly ventilator support, with limited access to existing facilities due to high costs. Hence, the case fatality rate of adult tetanus remains high. Women and children are protected because of regular and booster immunization strategies implemented around the world. However, men are disproportionately affected by tetanus. Booster dosage based on the World Health Organization (WHO)-recommended schedule should be given to eligible children and adolescent boys. In addition, tetanus vaccination needs to be promoted among adults in vulnerable jobs. Functional strategies could help pave the way toward the protection of all against tetanus.

## Introduction

Tetanus, caused by the toxin of the spore-forming bacterium *Clostridium tetani*, is now considered a disease of low- and middle-income countries (LMICs). Due to the widespread presence of spores in the environment, eradicating the disease is practically impossible [1]. Therefore, the World Health Organization (WHO) took strategic steps to eliminate tetanus instead of eradicating it. As a part of that initiative, vaccination strategies to eliminate neonatal tetanus (NT) and maternal tetanus (MT)/tetanus in women of reproductive age commenced approximately 2 decades ago. Subsequently, several milestones were achieved, particularly in the reduction of tetanus-inflicted neonatal and maternal deaths [2].

## Tetanus is still a problem in developing countries

Although vaccination has considerably reduced the global burden of tetanus, death and disability related to tetanus remain a public health problem in South Asia and sub-Saharan Africa. The Global Disease Burden (GBD) studies suggested that, as of 2017, approximately 82% of all tetanus cases in the world were reported from the aforementioned regions, along with an estimated 77% of the total tetanus deaths [3]. However, there could be a substantial underreporting of tetanus cases in GBD estimates compared with the actual counts [1]. Several reasons could be responsible for this low reporting, including one, no diagnostic tests are available to confirm tetanus, and, therefore, diagnosis depends on the identification of the clinical syndrome associated with tetanus reliant on clinicians’ expertise; two, LMICs are now better equipped to care for persons with acute respiratory failure, although the cause of the critical illness may not be diagnosed; and three, only childhood tetanus is considered reportable [1]. In addition, most surveys include only tetanus-associated deaths in their calculation, leaving the recovered cases uncounted. The majority of reports on adult tetanus in these regions are published in the form of case series or retrospective reviews of hospital records originating from specialized tertiary hospitals or teaching hospital settings [4].

Tetanus is characterized by a high case fatality rate. A meta-analysis revealed that the case fatality of tetanus among adults is still very high, with a rate of approximately 43.2% (95% CI 36.9% to 49.5%) [4]. To prevent death and aid recovery, tetanus management aims to control spasms and maintain cardiorespiratory stability. Wound debridement, neutralization of toxins, eradication of locally proliferating bacteria using antibiotics, and supportive care are the mainstays of treatment [5]. However, despite adequate initial care, many patients require ventilator support, which is scarce and not readily available in many LMICs. Additionally, private intensive care facilities are unaffordable for an average-income population due to high costs where care is not paid for through insurance policies [1,4]. Hence, preventing tetanus would be the most cost-effective approach to reducing the incidence of adult tetanus and its associated deaths.

## Men are particularly vulnerable to tetanus disease

**Fig 1:**
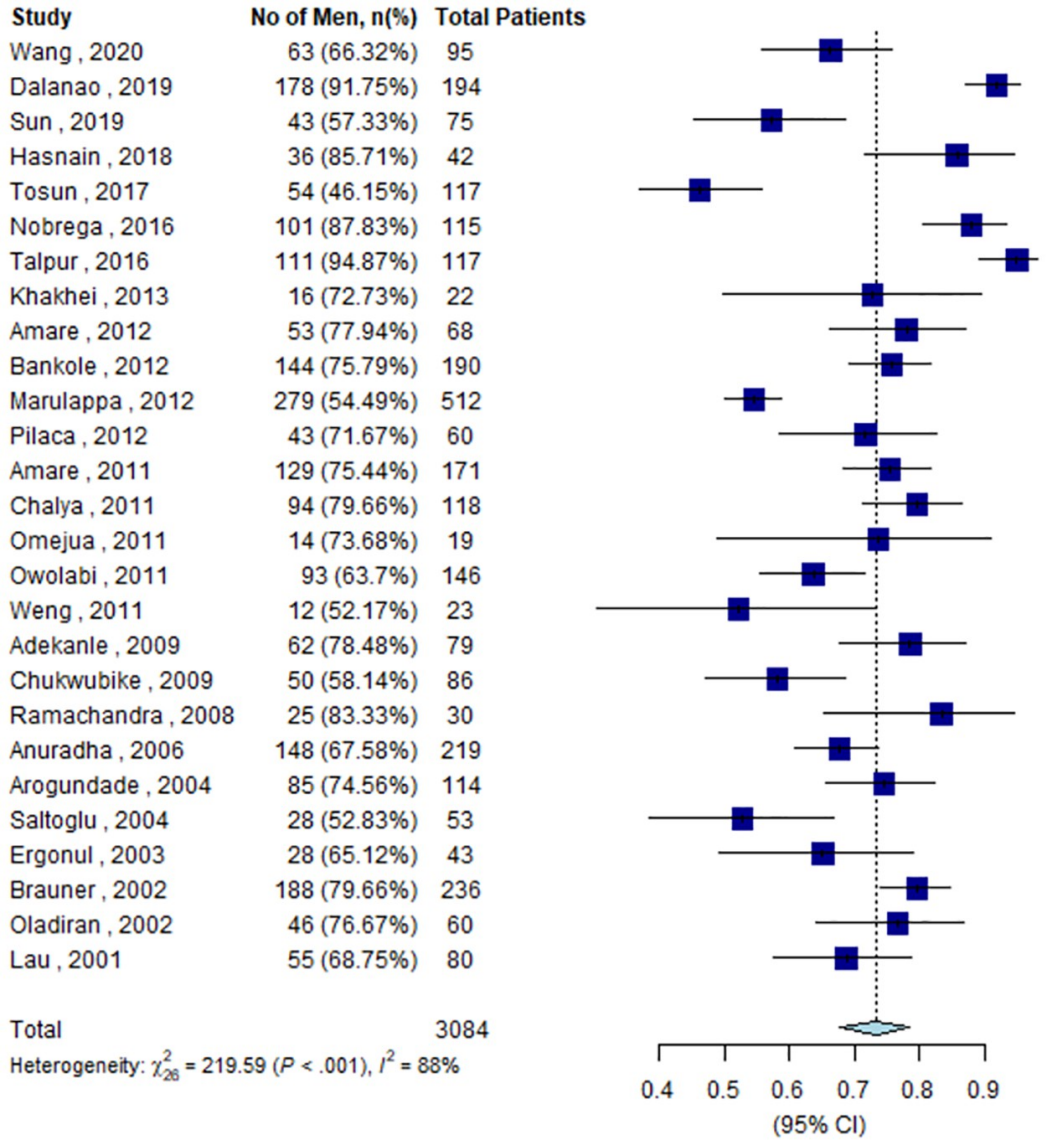
Forest plot showing a pooled proportion of men (73.42%; 95% CI: 67.71–78.45) among adult tetanus cases. The proportion was pooled using the random effect model and inverse variance method. A bibliography of the selected articles is provided in S1 File.

According to the policy recommendations, updated in 2017 by WHO, all populations worldwide should be vaccinated against tetanus. To achieve extended immunity throughout adulthood, WHO recommends 3 booster dosages of tetanus toxoid-containing vaccine (TTCV) between the ages of 12 to 23 months, 4 to 7 years, and 9 to 15 years after the primary infant series [2]. The primary series includes 3 doses of TTCV that should be administered starting at 6 weeks, with a minimum interval of 4 weeks between doses. The entire series should be completed by 6 months of age. These doses are usually integrated into the national immunization programs of most countries in combination with vaccines against one or more of the following diseases: diphtheria, pertussis, hepatitis B, *Haemophilus influenzae* type B, and polio. However, many LMICs do not have booster dosages in their current vaccination schedules [2]. However, to extend the coverage to women of childbearing age, most countries provide a 1-, 2-, or 5-dose schedule based on previous exposure to tetanus toxoid in accordance with the WHO guidelines [6]. However, adult males often remain unprotected if childhood immunization programs are missed and if there is no provision of the latter 3 booster doses in the schedule. This is also reflected in published studies, as we estimated the pooled proportion of men among adult tetanus patients admitted to hospitals between 2001 and 2020 to be 73.42% (95% CI: 67.71 to 78.45) (Fig 1). The majority of these studies (S1 File) are from LMICs, where men are frequently involved in jobs with a high risk of contracting tetanus, such as agriculture, butchery, day labor, fishing, and industrial work. Moreover, being a man is a significant predictor of case fatality among adults with tetanus [4].

The high prevalence of males among adult tetanus cases points to a lower-than-expected vaccination rate and higher occupational exposure [7,8]. While the need for vaccination throughout the life course is increasingly felt across nations, incorporating booster immunization requires special efforts [2]. Despite that, the remarkable success in implementing the Expanded Programme on Immunization [9] and the recent nationwide COVID-19 vaccination [10] by the Government of Bangladesh shows that well-coordinated and effective programs can take that challenge if the necessity demands action.

## Conclusion and recommendations

As remarkable success was achieved in reducing maternal and neonatal tetanus throughout the world, lowering tetanus among adult men is possible. Planning booster dosages among boys, adolescents, and men, such as those implemented for adolescent girls and women, has the potential to prevent thousands of tetanus-related deaths [11] around the globe. Moreover, families dependent solely on the income of men who do high-risk jobs for their family could be saved from economic and psychological breakdown due to tetanus-related death of their breadwinner through this approach. Additionally, this could contribute to decreasing the economic burden of a country. As tetanus vaccines are widely available at a relatively low cost, authorities in LMICs could plan booster vaccination programs at WHO-recommended age periods [2] within the range of their fiscal budgets or with help from global partners. Policy makers should communicate the necessity of preventing tetanus and explain the economic, social, and health benefits of the approach. Campaigns to raise awareness and promote vaccination among high-risk adult groups through appropriate health education programs and through dissemination of information using print, electronic, and social media could also help to maintain a high uptake of vaccines [12]. With proper education and realistic strategies, we can hope to protect nearly everyone from the never-ending threat of *Clostridium tetani*.

## Supporting information

S1 File Bibliography of the studies used to estimate the proportion of male adult tetanus patients. (DOCX)
