# Supplementary material for: The road towards protection of all against tetanus
Source: PLoS Negl Trop Dis. 2023 Sep 21;17(9):e0011611. doi: 10.1371/journal.pntd.0011611 (PMC10513208; doi:10.1371/journal.pntd.0011611)
Supplement: S1 File — (DOCX) [file pntd.0011611.s001.docx]

Studies were searched using the keywords “adult tetanus” in PubMed, Google Scholar and manually through the bibliography of the selected studies. Supplementary table 1 shows some details of the studies included.

**Supplementary table 1. Characteristics of the studies used for pooling proportion of male**

| Serial | Author | Year | Country | Location | Duration | Sample | Rural/ Urban | Design |
| --- | --- | --- | --- | --- | --- | --- | --- | --- |
|  | Wang et al[1] | 2020 | China | Fujian Province (5 hospitals) | January 2008 – December 2008 | Total: 95  Male: 63  Female: 32 | Unknown | Multicenter Retrospective |
|  | Dalanao et al[2] | 2019 | Philippines | Cagayan Valley Medical Center | October 2012 – September 2018 | Total: 194  Male: 178  Female: 16 | Rural: 172  Urban: 22 | Cross sectional |
|  | Sun et al[3] | 2019 | China | Jiangsu Province;  Nantong University Affiliated Hospitals | January 2005 – January 2017 | Total: 75  Male: 43  Female: 32 | Unknown | Retrospective |
|  | Hasnain et al[4] | 2018 | Bangladesh | Mymensingh District;  Surya Kanta Hospital | 2014 – 2015 | Total: 42  Male: 36  Female: 6 | Unknown | Retrospective case series |
|  | Tosun et al[5] | 2017 | Multi-country | 25 Medical Centers associated with Infectious Disease International Initiative | Not given | Total: 117  Male: 54  Female: 63 | Unknown | Multicenter Retrospective |
|  | Nobrega et al[6] | 2016 | Brazil | Fortaleza, Northeastern Brazil;  Hospital São José de Doencas Infecciosas | May 2003 – December 2013 | Total: 115  Male: 88  Female: 27 | Unknown | Retrospective |
|  | Talpur et al[7] | 2016 | Pakistan | Hyderabad;  Liaquat University Hospital, Wali Bhai Rajputana Hospital & MK Hospital | November 2009 – October 2015 | Total: 117  Male: 111  Female: 6 | Unknown | Descriptive Observational |
|  | Khakheli et al[8] | 2013 | Pakistan | Peoples University of Medical and Health Sciences Hospital | January 2010 – December 2012 | Total: 24  Male: 16  Female: 6 | Unknown | Descriptive case series |
|  | Amare et al[9] | 2012 | Ethiopia | Tikur Anbessa Hospital | June 2001 – May 2009 | Total: 68  Male: 53 Female: 15 | Unknown | Retrospective |
|  | Bankole et al[10] | 2012 | Nigeria | Lagos University Hospital | January 2000 – December 2009 | Total: 190  Male: 144  Female: 46 | Unknown | Retrospective Descriptive |
|  | Marulappa et al[11] | 2012 | India | Mysore, Karnataka;  Epidemic Disease (ED) Hospital | January 2001 – December 2010 | Total: 512  Male: 379  Female: 133 | Rural: 271  Urban: 25 | Retrospective |
|  | Pilaca et al[12] | 2012 | Albania | University Hospital Centre “Mother Theresa” of Tirana, Regional Hospital of Shkodra, and Regional Ho- spital of Korça | 1984 – 2002 | Total: 60  Male: 43  Female: 17 | Rural: 40  Urban: 20 | Retrospective |
|  | Amare et al[13] | 2011 | Ethiopia | Jimma University Teaching Hospital (JUTH) | July 1996 – August 2009 | Total: 171  Male: 129  Female: 42 | Unknown | Retrospective |
|  | Chalya et al[14] | 2011 | Tanzania | Bugando Medical Centre | January 2001 – December 2010 | Total: 102  Male: 94  Female: 8 | Unknown | Retrospective |
|  | Omejua et al[15] | 2011 | Nigeria | Nnewi;  Nnamdi Azikiwe University Teaching Hospital (NAUTH) | January 2005 – December 2009 | Total: 19  Male: 14  Female: 5 | Rural: 10  Urban: 9 | Retrospective |
|  | Owolabi et al[16] | 2011 | Nigeria | Northwestern Nigeria;  Aminu Kano Teaching Hospital (AKTH),  Murtala Muhammad Specialist Hospital (MMSH) | January 2004 – December 2009 | Total: 126  Male: 93  Female: 33 | Unknown | Retrospective review |
|  | Weng et al[17] | 2011 | Taiwan | Chang-Gung Memorial Hospital, Lin-Kou (CGMHLK) after | January 1996 – July 2005 | Total: 23  Male: 12  Female: 11 | Unknown | Retrospective review |
|  | Adekanle et al[18] | 2009 | Nigeria | Federal Medical Centre Owo, St Louis Catholic Hospital Owo | January 1997 - December 2006 | Total: 71  Male: 62  Female: 17 | Unknown | Review of medical records |
|  | Chukwubike et al[19] | 2009 | Nigeria | University of Port Harcourt Teaching Hospital (UPTH) | January 1996 – December 2005 | Total: 86  Male: 50  Female: 36 | Unknown | Retrospective case-note review |
|  | Ramachandra et al[20] | 2008 | India | Manipal;  Kasturba medical college hospital | January 2000 – December 2005 | Total: 30  Male: 25  Female: 5 | Unknown | Retrospective review of medical records |
|  | Anuradha [21] | 2006 | India | New Delhi;  Maulana Azad Medical College and associated Lok Nayak Hospital | January 1998 – December 2000 | Total: 219  Male: 148  Female: 69 | Unknown | Retrospective |
|  | Arongundade et al[22] | 2004 | Nigeria |  | 1992 – 2001 | Total: 114  Male: 85  Female: 29 | Unknown | Review of case records |
|  | Saltoglu et al[23] | 2004 | Turkey | Adana; Cukurova University Hospital | January 1994 – July 2000 | Total: 53  Male: 28  Female: 25 | Rural: 41  Urban: 12 | Retrospective |
|  | Ergonul et al[24] | 2003 | Turkey | Anakara Numune Training and Research Hospital | 1990 – 2000 | Total: 43  Male: 28  Female: 15 | Unknown | Retrospective |
|  | Brauner et al[25] | 2002 | Brazil | Hospital Nossa Senhora da Conceição, Hospital de Clinicas de Porto Alegre | October 1981 – March 2001 | Total: 236  Male: 188  Female: 48 | Unknown | Concurrent cohort study |
|  | Oladiran et al[26] | 2002 | Nigeria | Baptist Medical Centre of Ogbomoso (BMCO) | January 1995 – December 1999 | Total: 60  Male: 46  Female: 14 | Unknown | Retrospective study |
|  | Lau et al[27] | 2001 | Malaysia | Sarawak General Hospital | January 1999 – September 1999 | Total: 22  Male: 15  Female: 7 | Unknown | Retrospective review of records |

**Reference**

1. Wang X, Yu R, Shang X, Li J, Gu L, Rao R, et al. Multicenter Study of Tetanus Patients in Fujian Province of China: A Retrospective Review of 95 Cases. Biomed Res Int. 2020;2020. doi:10.1155/2020/8508547

2. Dalanao EJG, Valencia JCB. Clinical profile and prognostic factors of mortality among adult tetanus patients in cagayan valley medical center: A cross-sectional study. Phillippine J Intern Med. 2019;57: 200–208.

3. Sun C, Zhao H, Lu Y, Wang Z, Xue W, Lu S, et al. Prognostic factors for generalized tetanus in adults: A retrospective study in a Chinese hospital. Am J Emerg Med. 2019;37: 254–259. doi:10.1016/j.ajem.2018.05.039

4. Hasnain MG, Maruf S, Nath P, Anuwarul A, Ahmed MNU, Chowdhury IH, et al. Managing severe tetanus without ventilation support in a resource-limited setting in Bangladesh. Am J Trop Med Hyg. 2018;99: 1234–1238. doi:10.4269/ajtmh.18-0180

5. Tosun S, Batirel A, Oluk AI, Aksoy F, Puca E, Bénézit F, et al. Tetanus in adults: results of the multicenter ID-IRI study. Eur J Clin Microbiol Infect Dis. 2017;36: 1455–1462. doi:10.1007/s10096-017-2954-3

6. Nóbrega MVD da, Reis RC, Aguiar ICV, Queiroz TV, Lima ACF, Pereira EDB, et al. Patients with severe accidental tetanus admitted to an intensive care unit in Northeastern Brazil: clinical–epidemiological profile and risk factors for mortality. Brazilian J Infect Dis. 2016;20: 457–461. doi:10.1016/j.bjid.2016.06.007

7. Talpur AA, Channar KA, Balouch TA, Kumar B, Jamal A. Patterns of Morbidity and Mortality Among Tetanus Patients. Med Channel. 2016;22: 6–12.

8. Khakheli MS, Khuhro BA, Jamali AH. Tetanus: Still a killer in adults. Anaesthesia, Pain Intensive Care. 2013;17: 149–153.

9. Amare A, Melkamu Y, Mekonnen D. Tetanus in adults: Clinical presentation, treatment and predictors of mortality in a tertiary hospital in Ethiopia. J Neurol Sci. 2012;317: 62–65. doi:10.1016/j.jns.2012.02.028

10. Bankole IA, Danesi MA, Ojo OO, Okubadejo NU, Ojini FI. Characteristics and outcome of tetanus in adolescent and adult patients admitted to the Lagos University Teaching Hospital between 2000 and 2009. J Neurol Sci. 2012;323: 201–204. doi:10.1016/j.jns.2012.09.017

11. Marulappa VG, Manjunath R, Mahesh N, Maligegowda L. A ten year retrospective study on adult Tetanus at the epidemic disease (ED) hospital, Mysore in Southern India: A review of 512 cases. J Clin Diagnostic Res. 2012;6: 1377–1380. doi:10.7860/JCDR/2012/4137.2363

12. Pilaca AS, Beqiri AI, Ndreu AH, Puca ES, Pepa AK, Elezi FM. Factors affecting the prognosis of Albanian adult patients with generalized tetanus. G Chir. 2012;33: 105–9. Available: http://www.ncbi.nlm.nih.gov/pubmed/22668526

13. Amare A, Yami A. Case-fatality of adult Tetanus at Jimma University Teaching Hospital, Southwest Ethiopia. Afr Health Sci. 2011;11: 36–40.

14. Chalya PL, Mabula JB, Dass RM, Mbelenge N, Mshana SE, Gilyoma JM. Ten-year experiences with Tetanus at a Tertiary hospital in Northwestern Tanzania: A retrospective review of 102 cases. World J Emerg Surg. 2011;6: 2–9. doi:10.1186/1749-7922-6-20

15. Omejua E, Nwosu N, Onah S, Chukwurah S. A 5-Year Review of Tetanus Cases Among Adults in a Tertiary Hospital in South East Nigeria. Afrimedic J. 2011;2: 6–11. Available: https://www.ajol.info/index.php/afrij/article/view/86295/76122

16. Owolabi LF, Habib AG, Nagoda M. Predictors of mortality among adult tetanus patients in Northwestern Nigeria. Neurol Asia. 2011;16: 199–203.

17. Weng WC, Huang WY, Peng TI, Chien YY, Chang KH, Ro LS, et al. Clinical characteristics of adult tetanus in a Taiwan medical center. J Formos Med Assoc. 2011;110: 705–710. doi:10.1016/j.jfma.2011.09.007

18. Adekanle O, Ayodeji OO, Olatunde LO. Tetanus in a rural setting of south-western Nigeria: A ten-year retrospective study. Libyan J Med. 2009;4: 78–80. doi:10.4176/081125

19. Chukwubike OA, God’Spower AE. A 10-year review of outcome of management of tetanus in adults at a Nigerian tertiary hospital. Ann Afr Med. 2009;8: 168–172. doi:10.4103/1596-3519.57239

20. Ramachandra L, Shobha K, Kannan PA. A Retrospective Clinical Study of Factors Affecting Tetanus. internet J Microbiol. 2008;7: 1–6.

21. Anuradha S. Tetanus in adults - A continuing problem: An analysis of 217 patients over 3 years from Delhi, India, with special emphasis on predictors of mortality. Med J Malaysia. 2006;61: 7–14.

22. Arogundade FA, Bello IS, Kuteyi EA, Akinsola A. Patterns of presentation and mortality in tetanus: a 10-year retrospective review. Niger Postgrad Med J. 2004;11: 58–63. doi:10.4103/1117-1936.175147

23. Saltoglu N, Tasova Y, Midikli D, Burgut R, Dündar IH. Prognostic factors affecting deaths from adult tetanus. Clin Microbiol Infect. 2004;10: 229–233. doi:10.1111/j.1198-743X.2004.00767.x

24. Ergonul O, Erbay A, Eren S, Dokuzoguz B. Analysis of the case fatality rate of tetanus among adults in a tertiary hospital in Turkey. Eur J Clin Microbiol Infect Dis. 2003;22: 188–190. doi:10.1007/s10096-003-0904-8

25. Brauner J, Vieira RS, Bleck T. Changes in severe accidental tetanus mortality in the ICU during two decades in Brazil. Intensive Care Med. 2002;28: 930–935. doi:10.1007/s00134-002-1332-4

26. Oladiran I, Meier DE, Ojelade AA, OlaOlorun DA, Adeniran A, Tarpley JL. Tetanus: Continuing problem in the developing world. World J Surg. 2002;26: 1282–1285. doi:10.1007/s00268-002-6497-z

27. Lau LG, Kong KO, Chew PH. A ten-year retrospective study of tetanus at a General Hospital in Malaysia. Singapore Med J. 2001;42: 346–350.
